# Diabetes Mellitus Diagnosis and Screening in Australian General Practice: A National Study

**DOI:** 10.1155/2022/1566408

**Published:** 2022-03-23

**Authors:** Mingyue Zheng, Carla De Oliveira Bernardo, Nigel Stocks, David Gonzalez-Chica

**Affiliations:** ^1^Discipline of General Practice, Adelaide Medical School, The University of Adelaide, Adelaide, Australia; ^2^School of Health and Rehabilitation, Chengdu University of Traditional Chinese Medicine, Chengdu, China; ^3^Australian Partnership for Preparedness Research on Infectious Disease Emergencies (APPRISE) Centre of Research Excellence, NHMRC, Adelaide, Australia; ^4^EMPOWER: Health Systems, Adversity and Child Well Being Centre of Research Excellence, NHMRC, Adelaide, Australia; ^5^Adelaide Rural Clinical School, The University of Adelaide, Adelaide, Australia

## Abstract

**Aims:**

To investigate the epidemiology of diabetes diagnosis and screening in Australian general practice.

**Methods:**

Cross-sectional study using electronic health records of 1,522,622 patients aged 18+ years attending 544 Australian general practices (MedicineInsight database). The prevalence of diagnosed diabetes and diabetes screening was explored using all recorded diagnoses, laboratory results, and prescriptions between 2016 and 2018. Their relationship with patient sociodemographic and clinical characteristics was also investigated.

**Results:**

Overall, 7.5% (95% CI 7.3, 7.8) of adults had diabetes diagnosis, 0.7% (95% CI 0.6, 0.7) prediabetes, and 0.3% (95% CI 0.3, 0.3) unrecorded diabetes/prediabetes (elevated glucose levels without a recorded diagnosis). Patients with unrecorded diabetes/prediabetes had clinical characteristics similar to those with recorded diabetes, except for a lower prevalence of overweight/obesity (55.5% and 69.9%, respectively). Dyslipidaemia was 1.8 times higher (36.2% vs. 19.7%), and hypertension was 15% more likely (38.6% vs. 33.8%) among patients with prediabetes than with diabetes. Diabetes screening (last three years) among people at high risk of diabetes was 55.2% (95% CI 52.7, 57.7), with lower rates among young or elderly males.

**Conclusions:**

Unrecorded diabetes/prediabetes is infrequent in Australian general practice, but prediabetes diagnosis was also lower than expected. Diabetes screening among high-risk individuals can be improved, especially in men, to enhance earlier diabetes diagnosis and management.

## 1. Introduction

Diabetes mellitus is a major global health problem and one of the fastest-growing chronic conditions [1]. In Australia, the age-standardised ratio of self-reported diabetes has increased from 3.3% in 2001 to 4.4% in 2017-2018 [2]. However, diabetes is not always medically diagnosed. Globally, it is estimated that one in two people living with diabetes is unaware of their condition [3]. Several nationwide studies have investigated the actual magnitude of undiagnosed diabetes, either using electronic health records (EHRs) [4] or through laboratory tests used as part of national surveys [5–7]. The prevalence of unreported diabetes in the United States (US) was estimated at 0.9% in 1988-1994 and 1.2% in 2011-2014 [5], while a French national study found a prevalence of 1.7% in 2014-2016 [7].

Moreover, prediabetes (a condition where the glycaemic parameters are above normal but below the threshold for diabetes [8]) increases the burden of diabetes, with a conversion rate to diabetes of 5%-10% per year [9]. Globally, the estimated prevalence of prediabetes was 7.5% in 2019 (~374 million people) and is projected to reach 8.6% (~548 million people) by 2045 [3]. In Australia, prediabetes affects 3.1% of adults [10]. Undiagnosed prediabetes is an additional concern, as these individuals are at a higher risk of complications, including chronic kidney disease (CKD), diabetic retinopathy, and macrovascular disease [11].

Therefore, early detection of prediabetes and diabetes is crucial for appropriate management and prevention of disease progression [12, 13]. According to the Australian Guidelines for Preventive Activities in General Practice [14], regular (within three years) diabetes screening is recommended for those with a clinical history of gestational diabetes mellitus or polycystic ovary syndrome (PCOS) and those treated with antipsychotics or at higher risk of cardiovascular disease (CVD). Screening among these individuals should be performed regularly, either through fasting blood glucose (FBG) or haemoglobin A1c (HbA1c) tests [14–17]. Beyond these groups, noninvasive and straightforward tools such as the Australian Type 2 Diabetes Risk (AUSDRISK) Assessment Tool questionnaire have been developed to identify other individuals at risk of diabetes who require further assessment [11, 18, 19]. For example, the AUSDRISK is a questionnaire that scores the probability of a person developing diabetes mellitus within five years or with undiagnosed diabetes [20]. People with a score ≥ 12 points should then have their blood glucose levels tested [14].

Diabetes screening in a primary care setting is widely recommended, considering that more than 83% of the population use these services every year [21], making it an ideal environment for early diabetes diagnosis and management. Despite this, population-based national studies or data on whether diabetes screening activities are being performed in primary care following current recommendations are scarce [18]. In this sense, EHRs generated by general practitioners (GPs) during medical appointments represent a unique data source for investigating the prevalence of diabetes and prediabetes diagnoses, screening activities, and management of these conditions. In addition, data extracted from EHR databases has been found a cost-effective method for exploring different health outcomes with appropriate accuracy [4, 22–25].

In Australia, EHRs have been used in the last decade to estimate the burden of various chronic conditions, but only a few have focused on diabetes [24, 26–30]. Data from the Bettering the Evaluation and Care of Health program (BEACH), a national study of general practice activity that included GP-reported data (Nov/2012 to Mar/2016), showed a prevalence of type 2 diabetes of 9.6% among adults [31]. In Victoria, the Outcome Health's Population Level Analysis & Reporting (POLAR) used recorded pathology results to explore the prevalence of type 2 diabetes among adults (4.9%), showing results comparable to Australian population-based estimates (5.2%) and with a similar distribution according to sociodemographic characteristics [24]. Finally, MedicineInsight, a large general practice Australian database, has been used to explore diabetes mellitus, prescriptions, and associated comorbidities [26, 27, 29]. However, none of these studies investigated prediabetes, the magnitude of undiagnosed diabetes/prediabetes, or diabetes screening at a national level.

Therefore, this study is aimed at (1) identifying the prevalence of recorded or unrecorded diabetes and prediabetes among adults in Australian general practice, (2) comparing these groups according to sociodemographic and clinical characteristics, and (3) assessing if diabetes screening was more likely among people at high risk of diabetes.

## 2. Material and Methods

### 2.1. Data Source

This is a cross-sectional study using MedicineInsight, a large national general practice database managed by NPS MedicineWise. The database contains deidentified EHRs from more than 650 general practices (8.2% of all practices in the country) and over 2,700 GPs from all Australian states and regions. This ongoing longitudinal database includes practices varying in size, billing methods, and type of services [32]. Details of the data collection process and characteristics of the database have been published elsewhere [33].

Routinely collected data available in MedicineInsight include sociodemographic (i.e., gender, year of birth, and postcode of residence) and clinical data (i.e., diagnoses, reasons for consultation, and smoking status), prescribed medications and reasons for these prescriptions, laboratory/pathology test results (e.g., blood glucose levels and lipid profile), and clinical measurements (e.g., blood pressure, weight, and height).

### 2.2. Study Population

Following recommendations for improving data quality [23, 34, 35], only data from practices established at least two years before the end of the analysis period and without interruptions in data greater than six weeks was included in the study. Moreover, analysis was restricted to adults (18+ years) considered “regular” patients (at least three consultations in any two consecutive years (i.e., “active” patient, as defined by the Royal Australian College of General Practitioners to identify frequent users of the service and for reporting purposes) [36] and at least one consultation in each of these two years) and attending a MedicineInsight general practice between Jan/2016 and Dec/2018. Our definition of “regular” patients takes into account recommendations for improving diagnosis accuracy when using EHR and the specificities of diabetes diagnosis that requires multiple encounters to request the tests and discuss diagnosis/management with the patient [23, 34, 35]. Administrative contacts (e.g., “email,” “reminder,” “letter,” and “filling forms”) were excluded as encounters.

### 2.3. Data Extraction

Different fields in MedicineInsight (i.e., “diagnosis,” “reason for encounter,” and “reason for prescription”) were searched to identify patients with a recorded diagnosis of diabetes mellitus (either type 1 or type 2) or prediabetes (also recorded as impaired glucose tolerance or impaired fasting glucose), using standard clinical terminology, abbreviations, and misspellings of these words. The algorithm for data extraction also identified all prescriptions of insulin (Anatomical Therapeutic Chemical Classification (ATC) code A10A) and/or oral antidiabetic medications (ATC code A10B: metformin, glibenclamide, gliclazide, glimepiride, glipizide, acarbose, pioglitazone, alogliptin, linagliptin, saxagliptin, sitagliptin, vildagliptin, dulaglutide, exenatide, dapagliflozin, empagliflozin, and ertugliflozin) during the study period. FBG (mmol/L), random blood glucose (mmol/L), HbA1c (mmol/L or %) and 2-hour oral glucose tolerance test (OGTT) (mmol/L), and date of these tests were obtained from all recorded laboratory results using Logical Observation Identifiers Names and Codes [32]. The use of medications and laboratory results combined with recorded diabetes diagnosis improves the data quality and accuracy of estimates based on EHRs [23].

**Figure 1:**
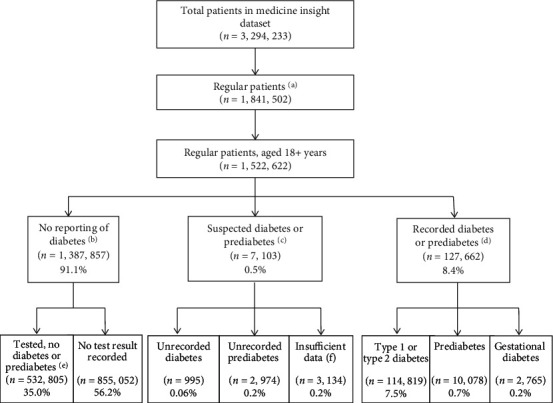
Flowchart of the distribution of patients included in the study, their screening status, and diagnosis of diabetes or prediabetes in Australian general practice. MedicineInsight, 2016-2018. (a) At least three consultations in two consecutive years and at least one in each year. (b) No recording of diabetes, either as a diagnosis, reason for encounter, reason for prescription, or receiving an antidiabetic medication over the three-year period. (c) One or more positive laboratory results for diabetes or prediabetes (details in Supplementary Table 1) but no recorded diagnosis of diabetes or prediabetes or prescription of antidiabetic medication. (d) Diagnosis (diabetes, prediabetes, and gestational diabetes) recorded in at least two different occasions either as a diagnosis, reason for encounter, reason for prescription, or patient was prescribed antidiabetic medication, or the diagnosis was recorded only once but the patient had a positive laboratory result consistent with diabetes or prediabetes. (e) At least one laboratory test recorded, all results negative for diabetes or prediabetes. (f) Only one positive blood test for diabetes or prediabetes recorded, but no recorded diagnosis or prescription for diabetes/prediabetes.

Patients were considered as having diabetes when (1) diabetes diagnosis was recorded (“diagnosis,” “reason for encounter,” and “reason for prescription”) on two different occasions between 2016 and 2018, or (2) a patient was prescribed antidiabetic medication (ATC A10A or A10B, metformin considered only in the absence of PCOS diagnosis), or (3) diabetes diagnosis was recorded only once but the patient had in the preceding 24 months at least one laboratory result (FBG, HbA1c, or OGTT) above the threshold for diabetes diagnosis [14] (Supplementary Table 1). A similar approach was used to identify patients with prediabetes, considering a combination of (1) two records of prediabetes diagnosis or (2) only one record plus metformin prescription (i.e., in the absence of PCOS or diabetes diagnosis) or laboratory results consistent with impaired glucose levels. Patients with at least two laboratory results above recommended thresholds (either FBG or HbA1c) and/or a positive OGTT, but without any record of diabetes or prediabetes diagnosis or any prescribed antidiabetic medication were classified as “unrecorded” diabetes or “unrecorded” prediabetes. When only one abnormal FBG or HbA1c laboratory result was recorded, but not diabetes/prediabetes diagnosis was recorded or antidiabetic medication prescribed, patients were classified as “insufficient data” (Figure 1 and Supplementary Table 1).

Additional data extracted from the dataset included risk factors for diabetes (age 40+ years and overweight/obesity, AUSDRISK score ≥12 points, clinical history of CVD (including ischaemic heart disease and stroke), gestational diabetes, PCOS, or current use of antipsychotics (ATC N05A; 2018 only)) and other clinical conditions related to diabetes or prediabetes (hypertension, dyslipidaemia, CKD, atrial fibrillation, and heart failure) [14]. Data extraction was performed based on algorithms used in previous studies [25, 30, 33]. Overweight/obesity diagnosis used records of these terms as a “diagnosis,” “reason for encounter,” or “reason for prescription,” and body mass index data (i.e., ≥25.0 kg/m^2^) recorded in the same fields or as a clinical measure in the “observation” field. The AUSDRISK score among patients without recorded diabetes diagnosis was calculated based on six of the 13 recommended variables: age, gender, Aboriginal status, smoking status, the antecedent of high blood glucose (i.e., FBG levels), and the prescription of antihypertensive medications (Supplementary Table 2) [20]. Vegetable or fruit intake, physical activity levels, a family history of diabetes, or waist circumference values were not used to estimate the AUSDRISK score as they are not consistently recorded in MedicineInsight [33]. Data extraction algorithms used in this study are available under request.

### 2.4. Outcomes and Covariates

The first investigated outcome was the prevalence of recorded diabetes, recorded prediabetes, and unrecorded diabetes/prediabetes, presented as a proportion of “regular” adult patients in the database. The second outcome was the prevalence of recorded diabetes screening (i.e., at least one laboratory result of any blood glucose test recorded between 2016 and 2018) among patients at high risk of diabetes (i.e., patients without a diabetes diagnosis, but with some of the conditions listed above, including prediabetes). Current guidelines recommend that individuals at high risk of diabetes should have their glucose levels checked at least every three years (every 12 months for prediabetes), preferably by testing FBG or HbA1c [14]. Diabetes screening was defined as having at least one recorded blood glucose test result (FBG, HbA1c, random levels, OGTT, or finger-prick test), irrespective of the reported value.

Covariates included patient data (gender (male and female), age (categorised as 18-29, 30-39, 40-49, 50-59, 60-69, 70-79, 80-89, and 90+ years), comorbidities, and median number of consultations) and practice data (practice remoteness (major cities, inner regional, or outer regional/remote) and Index of Relative Socioeconomic Advantage and Disadvantage (IRSAD, in quintiles)). IRSAD is a macroeconomic indicator of socioeconomic status based on postcodes and generated by the Australian Bureau of Statistics based on a range of census variables [37]. A higher IRSAD score indicates the practice is located in a more advantaged area. The investigated comorbidities included overweight/obesity, hypertension, dyslipidaemia, CKD, ischaemic heart disease, atrial fibrillation, heart failure, and stroke [14].

### 2.5. Statistical Analyses

All analyses were conducted in Stata MP 16.1 (StataCorp, Texas, USA), with the practice as a cluster, using robust standard errors and conditioned to the number of visits to the practice. The sociodemographic profile of those with unrecorded prediabetes/diabetes was compared to those with recorded diabetes or recorded prediabetes using Chi-square test. The same procedure was used to compare the prevalence of risk factors (i.e., overweight/obesity, hypertension, dyslipidaemia, and CKD) and coexisting CVD (i.e., ischaemic heart disease, atrial fibrillation, heart failure, and stroke) among those with recorded or unrecorded diabetes/prediabetes. The results were presented graphically with the corresponding 95% confidence intervals (95% CI).

The prevalence of diabetes screening among those at high risk of diabetes was estimated overall (at least one of these risk factors) and for each risk factor. Furthermore, to assess how screening was performed over the lifespan, the prevalence of diabetes screening according to age and gender was presented graphically, separately for those at high-risk (i.e., at least one risk factor) or not at high risk of diabetes. Differences in diabetes screening according to age, gender, and risk status were assessed using Chi-square tests.

This study followed the REporting of studies Conducted using Observational Routinely-collected health Data (RECORD) statement [35]. The independent MedicineInsight Data Governance Committee approved the study (protocol 2016-007). The Human Research Ethics Committee of the University of Adelaide exempted the study of an ethical review as it used only existing and nonidentifiable data.

## 3. Results

The sample included 1,522,622 “regular” patients aged 18+ years (41.9% males, mean age 49.8 ± 19.1 years) attending 544 general practices (Figure 1 and Table 1). The prevalence of recorded diabetes was 7.5% (95% CI 7.3, 7.8), recorded prediabetes 0.7% (95% CI 0.6, 0.7), and unrecorded diabetes/prediabetes 0.3% (95% CI 0.3, 0.3). Supplementary Figures 1 and 2 show the prevalence of these outcomes according to sociodemographic characteristics.

Table 1 shows that the median number of consultations was lower among those with recorded prediabetes than in the other two groups. The mean age of patients with unrecorded diabetes/prediabetes (68.5 ± 13.3 years) was higher than those with recorded diabetes (63.5 ± 15.6 years) or recorded prediabetes (60.3 ± 13.4 years). Still, the distribution according to gender, practice remoteness, and practice IRSAD quintile was similar. Supplementary Table 3 presents further details on these comparisons (i.e., proportions with the corresponding 95% CI).

**Figure 2:**
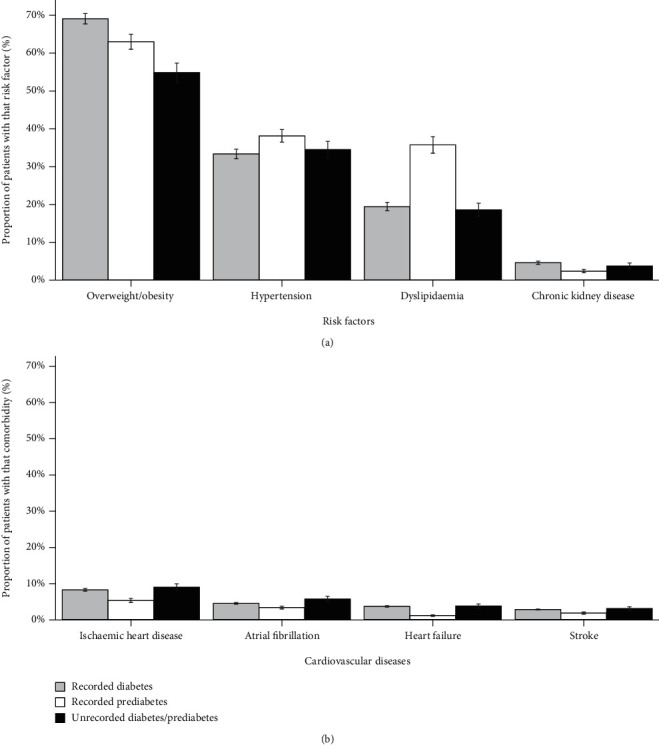
Prevalence of diabetes-related comorbidities ((a) risk factors for cardiovascular disease; (b) cardiovascular disease) among regular patients (aged 18+ years) with recorded diabetes, recorded prediabetes, and unrecorded diabetes/prediabetes (Australia, 2016-2018).

Figure 2 shows the prevalence of risk factors for CVD (Figure 2(a)) or established CVD (Figure 2(b)) according to diabetes/prediabetes diagnosis status. Overweight/obesity was the most prevalent risk factor, affecting 69.9% of patients with diabetes, 63.8% of those with prediabetes, and 55.5% of those with unrecorded diabetes/prediabetes. Dyslipidaemia was around twice higher (36.2% vs. 19.7%), and hypertension was 15% more likely (38.6% vs. 33.8%) among patients with prediabetes than with diabetes. In contrast, all cardiovascular conditions were less frequent among those with recorded prediabetes. Except for the lower prevalence of overweight/obesity, patients with unrecorded diabetes/prediabetes had a similar clinical profile to those with recorded diabetes.

Table 2 presents the results for diabetes screening among patients with no diabetes diagnosis. The prevalence of diabetes screening was 71% more likely among those with at least one risk factor for diabetes (55.2%, 95% CI 52.7, 57.7) than those not at high risk of diabetes (32.3%, 95% CI 30.5, 34.1). In addition, diabetes screening was slightly higher among those with a higher AUSDRISK score (61.3%), CVD (57.1%), or aged 40+ years and overweight/obese (56.6%). The lowest prevalence of diabetes screening was for those treated with antipsychotic (27.0%) or with prediabetes diagnosis (45.5%).

**Figure 3:**
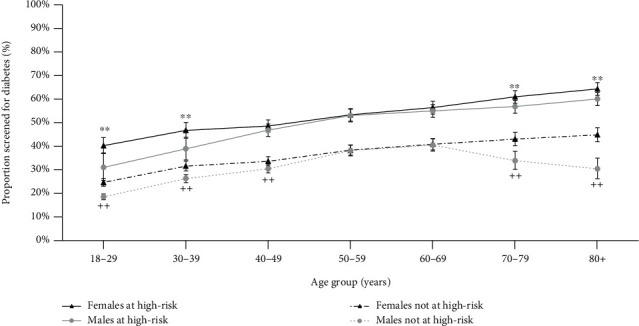
Prevalence of having a record of diabetes screening in males and females according to age and presence or not of risk factors for diabetes. *P* value for the difference between males and females at high risk: ^∗^<0.01 and ^∗∗^<0.001; *P* value for the difference between males and females not at high risk: ^+^<0.01 and ^++^<0.001.

The prevalence of diabetes screening according to gender, age, and presence of risk factors for diabetes is shown in Figure 3. Overall, the prevalence of diabetes screening increased with the age of the patients, but the association with gender varied across age groups. Diabetes screening was less frequent in younger males (18-39 years) than females, with a more pronounced difference among those at high risk of diabetes. However, gender differences were less evident among those aged 40-69 years, whether they were or were not at high risk of diabetes. After that age, diabetes screening was again less frequent in men, showing a decline among those not at high risk of diabetes.

## 4. Discussion

Five main findings can be highlighted based on our results. First, the prevalence and distribution of diabetes according to age and gender were consistent with national figures. Second, patients with prediabetes showed a higher prevalence of hypertension and dyslipidaemia than those with diabetes. Third, the prevalence of prediabetes diagnosis was lower than expected, but unrecorded diabetes/prediabetes was also infrequent. Fourth, the last finding probably underrepresents actual figures, as 45% of patients at high risk of diabetes were not screened for diabetes over three years. Those treated with antipsychotics had the lowest frequency of diabetes screening. Finally, diabetes screening increased with age and was lower in males. Still, the gender difference lessened among those aged 40-69 years, whether they were or were not at high-risk of diabetes.

According to Australian National Health Survey (NHS), the prevalence of diabetes among adults was 5.1% in 2011-2012 (combining self-reported and laboratory results) and 6.2% in 2017-2018 (self-reported data only) [10, 38]. The lower prevalence observed in the most recent NHS compared to our study (7.5%) may reflect the use of a community-based sample in that survey compared to people seeking medical care in MedicineInsight, as well as the use of self-reported data and misclassification error of those with undiagnosed diabetes [38].

Globally, it is estimated that one in two people living with diabetes does not know he/she has diabetes [3]. However, these proportions are lower in high-income countries. In the US, data from the National Health and Nutrition Examination Survey (NHANES, 2011-2014) showed that between 23% and 35% of people with diabetes were undiagnosed (using either FBG/HbA1c or 2-hour plasma glucose tolerance test, respectively) [12]. A French national survey conducted between 2014 and 2016 found that 23% of people living with diabetes were undiagnosed (FPG results), with a prevalence three times higher in males than females [7]. In Australia, data from the NHS in 2011-2012 showed that 18% of adults living with diabetes were undiagnosed (FPG and HbA1c results), increasing the estimated prevalence of diabetes from 4.2% (known diabetes) to 5.1% (total diabetes) [10].

According to our findings, once a patient has tested positive for diabetes or prediabetes, it is more likely their status will be updated in the EHRs (i.e., only 0.26% of adults had unrecorded diabetes/prediabetes). As well as reducing misclassification bias due to undiagnosed diabetes, another advantage of studies based on EHRs is that they can help monitor annual changes in the prevalence of diabetes and other chronic conditions [33].

Our results are slightly different from other Australian studies that used medical records. POLAR found 4.9% of adults attending practices in urban Victoria had diabetes in 2016 (recorded diagnosis only) [24]. Still, using GP-reported data, BEACH found 10.4% of adults in Australia had a diagnosis of diabetes (2012-2016) [31]. The discrepancy across studies is probably related to the different methodological approaches used to identify patients with diabetes.

In this regard, analyses based on EHR databases rely on proper data recording and data extraction. In our study, one result that is lower than expected is the prevalence of prediabetes (0.7% compared to 3.1% in the Australian NHS from 2011-2012) [10]. Most Australian general practices use automatic methods to download the laboratory results (Logical Observation Identifiers Names and Codes, values, date, and limits of the results) into the EHRs [32], making data extraction a less likely source of information bias. Nonetheless, four in ten patients at risk of diabetes had no record of a glucose test in the last three years, suggesting the prevalence of prediabetes and undiagnosed diabetes is higher than observed.

Current Australian guidelines recommend regular laboratory diabetes screening only for those at high risk of diabetes [14, 19]. Nonetheless, compliance with these recommendations was suboptimal, as one-half of individuals at increased risk of diabetes were screened for diabetes in three years (one-third among those not at high risk of diabetes). This finding is consistent with results from the NHANES in the US, where 46% of adults at high-risk of diabetes reported diabetes screening, compared to 30% among those for whom screening was not recommended [39]. In a recent South Australian survey including a population-based sample of individuals aged 35+ years, diabetes screening in the last 12 months was reported by 69% of those with cardiometabolic conditions, 75% of those with CVD and 51% of those with none of these conditions [40].

In our study, less than half of patients with prediabetes were screened for diabetes in the last 12 months, which is a concern, as the conversion rate to diabetes among them is 5%-10% per year [9, 14]. Moreover, patients with recorded prediabetes showed a higher prevalence of dyslipidaemia and hypertension than those with diabetes. The last finding is counterintuitive, as we expected a better metabolic profile among patients with prediabetes when compared to those with diabetes, as the former were younger (mean age of 60.3 vs. 63.5 years) and had a lower prevalence of obesity (63.8% vs. 69.9%). Moreover, a national cross-sectional study involving 69,974 middle-aged Chinese people showed the prevalence of dyslipidaemia was higher in patients with type 2 diabetes than with prediabetes (59.3% vs. 46.8%) [41]. It is possible the worst metabolic profile observed among patients with prediabetes resulted from different sources of error, including detection bias (i.e., GPs were more likely to test, diagnose, and/or record hypertension and dyslipidaemia to reduce diabetes progression; hypertension/dyslipidaemia diagnosis leading to the diagnosis of “asymptomatic” prediabetes), survival bias (i.e., patients with diabetes in the database represent “survivor” cases with a better metabolic profile), and/or underdiagnosis of patients with less complicated forms of prediabetes. Therefore, our findings require cautious interpretation, and further longitudinal studies using primary data collection would be necessary to verify these results.

An even lower screening rate was found for patients treated with antipsychotics, at just over a quarter in 2018, which is worrying as antipsychotics have severe effects on blood glucose levels [42]. Tests outside general practice (i.e., hospital or mental health services) are not captured in MedicineInsight, which may explain these lower numbers. However, a large retrospective cohort study in the US using comprehensive data of all performed tests (FBG or HbA1c, either in primary care or mental health services) found that only 30% of nondiabetic patients treated with antipsychotics were screened for diabetes over 12 months [43]. Moreover, that study also reported that patients that had visited a primary care doctor in addition to mental health services were twice more likely to be screened than those who did not. Another possible explanation for the lower screening rates among patients treated with antipsychotics in our study is their younger age (median 50 years and interquartile range 37-67 years) compared to those with other risk factors for diabetes (median 63 years and interquartile range 51-73 years). The lower prevalence of diabetes screening among younger individuals has been reported in other studies [39, 40, 43, 44].

Regardless of being at risk or not of diabetes, screening was lower among males, which is also consistent with previous studies [39, 43]. This finding is likely related to more frequent health-service seeking behaviour in females [45, 46]. Nonetheless, men and women aged 40-69 years showed similar diabetes screening rates, which may reflect the influence of current chronic disease screening programs in midlife (e.g., 45-49 Year Old Health Check program) [14, 47].

This study used a large national database including general practices from all states and geographic regions to provide a comprehensive profile of diabetes diagnosis and screening in Australia. The study design incorporated methodological recommendations from previous studies using large datasets to improve data quality [23, 34, 35].

However, this study is not free of limitations. First, data in MedicineInsight was recorded by GPs as part of their daily clinical activities, which may affect the completeness and accuracy of recorded data. Second, patients who visit multiple general practices or who are not “regular” patients may have had their blood glucose levels tested in other settings (e.g., hospitals or specialists) or not tested at all. This selection bias is an additional limitation that probably contributed to the low prevalence of prediabetes and unrecorded diabetes/prediabetes when compared to national figures. Third, due to ethical issues that restrict the access to fields with potentially identifiable information, it was not possible to get access to the “progress notes” of an appointment, which may contain relevant clinical data. Moreover, the accuracy of the extracted information is another limitation. This limitation is mitigated by data checking: compared to the original EHRs available at the participating practices, data extracted from MedicineInsight had a sensitivity of 89% and specificity of 100% in identifying patients with diabetes [25].

## 5. Conclusions

MedicineInsight represents a valuable resource for monitoring and providing a comprehensive diabetes diagnosis and diabetes screening profile in Australian general practice, considering that unrecorded diagnosis among those tested is uncommon. However, the rate of diabetes screening among patients at high risk of diabetes can be substantially improved, as these individuals have an average of five encounters per year with their GP. Specific interventions should target diabetes screening among patients with prediabetes and those treated with antipsychotics. National strategies such as the 45-49 Year Old Health Check program [47] seem to have reduced gender disparities for diabetes screening in midlife. Expanding that program to younger and older individuals at high risk of diabetes may be beneficial for improving early diagnosis and reducing further complications, especially in men.

## Figures and Tables

**Table 1:** Sociodemographic profile of the study population (regular patients aged 18+ years) according to diabetes diagnosis status (2016-2018).

Characteristics	All patients, aged 18+ years (%)	Recorded diabetes (%)	Recorded prediabetes (%)	Unrecorded diabetes/prediabetes (%)
Number of consultations in 2018, median (IQR)	3 (2-7)	7 (3-13)^b^^∗∗^	5 (3-10)^c^^∗∗^	7 (3-12)
Age, mean ± SD	49.8 ± 19.1	63.5 ± 15.6^b^^∗∗^	60.3 ± 13.4^c^^∗∗^	68.5 ± 13.3
Gender: males	41.9	52.2	54.8	53.7
*Age group*				
18-29	17.9	3.1^b^^∗∗^	1.5^c^^∗∗^	0.5
30-39	17.1	5.6^b^^∗∗^	6.2^c^^∗∗^	2.8
40-49	16.1	9.7^b^^∗∗^	13.6^c^^∗∗^	5.4
50-59	16.0	17.1^b^^∗∗^	23.8^c^^∗∗^	14.0
60-69	15.1	25.6^b^^∗^	29.4	27.5
70-79	11.2	24.8^b^^∗∗^	19.5^c^^∗∗^	29.6
80-89	5.5	12.4^b^^∗∗^	5.6^c^^∗∗^	17.1
90+	1.1	1.7^b^^∗∗^	0.4^c^^∗∗^	3.0
*Practice remoteness*				
Major cities	64.5	60.3	64.5	57.9
Inner regional	23.5	26.2	23.7	27.2
Outer regional/remote	12.0	13.5	11.8	14.9
*Practice IRSAD quintile^a^*				
Very high	25.3	19.1^b^^∗∗^	23.0	23.1
High	19.4	17.0	19.3	17.3
Middle	22.8	24.6	23.2	23.1
Low	16.3	18.3	16.2	15.9
Very low	15.5	20.3	17.6	20.1

IQR: interquartile range; SD: standard deviation; IRSAD: Index of Relative Socioeconomic Advantage and Disadvantage. ^a^IRSAD had 0.8% of missing data; high quintiles indicate greater advantage, and low quintiles indicate greater disadvantage. ^b^*P* value for the difference between people with recorded diabetes and unrecorded diabetes/prediabetes. ^c^*P* value for the difference between people with recorded prediabetes and unrecorded diabetes/prediabetes. ^∗^*P* < 0.01; ^∗∗^*P* < 0.001.

**Table 2:** Proportion of diabetes screening^a^ according to the presence or not of risk factors for diabetes. Regular patients aged 18+ years (*n* = 1,407,803).

Risk factor for diabetes	*N* ^a^	Screened for diabetes (2016-2018)		Consultations in 2018 median (IQR)
		*n* ^b^	% (95% CI)	
None of them	999,352	322,302	32.3 (30.5-34.1)	2 (1-5)
At least one risk factor	408,451	225,620	55.2 (52.7-57.7)	5 (2-10)
Aged 40+ years and overweight/obesity	300,939	170,352	56.6 (53.9-59.2)	5 (2-10)
AUSDRISK score ≥ 12	117,406	71,921	61.3 (58.8-63.7)	6 (3-11)
Prediabetes^c^	10,078	4,582	45.5 (42.8-48.2)	5 (3-10)
Cardiovascular disease	40,542	23,142	57.1 (54.4-59.7)	8 (3-14)
History of gestational diabetes mellitus	2,765	1,505	54.4 (49.7-59.1)	4 (2-9)
Polycystic ovary syndrome	6,253	2,885	46.1 (42.9-49.4)	3 (2-7)
Antipsychotics^c^	27,692	7,492	27.0 (25.3-28.8)	8 (4-16)

95% CI: 95% confidence interval; IQR: interquartile range; AUSDRISK: Australian Type 2 Diabetes Risk Assessment Tool. ^a^Regular patients aged 18+ years in each subgroup, excluding those with recorded diabetes diagnosis (*n* = 114,819). ^b^Patients with at least one record of any blood glucose test in the last three years (2016-2018). ^c^Patients with at least one record of any blood glucose test in the last 12 months (2018).

## Data Availability

Data used in this study was obtained from a third party (MedicineInsight) for this specific project and cannot be released. Information about MedicineInsight data and how they can be accessed is available on the website (https://www.nps.org.au/medicine-insight). The data extraction algorithms used in this study are available from the corresponding author upon request.

## Supplementary Materials

Supplementary 1 Supplementary Table 1: definitions of recorded diabetes, recorded prediabetes, and unrecorded diabetes/prediabetes.

Supplementary 2 Supplementary Table 2: calculation of the Australian Type 2 Diabetes Risk (AUSDRISK) Assessment Tool score using variables available in the MedicineInsight database.

Supplementary 3 Supplementary Table 3: sociodemographic profile of the study population (regular patients aged 18+ years) with 95% CI according to diabetes diagnosis status (2016-2018).

Supplementary 4 Supplementary Figure 1: prevalence of recorded diabetes (A), recorded prediabetes (B), and unrecorded diabetes/prediabetes (C) among all adults aged 18+ years, by age group and gender, Australia, 2016-2018.

Supplementary 5 Supplementary Figure 2: proportion of recorded diabetes, recorded prediabetes, and unrecorded diabetes/prediabetes among regular patients aged 18+ years, by gender (A), age (B), practice remoteness (C), and practice IRSAD (D), Australia, 2016-2018.

## References

[1] Bommer C., Sagalova V., Heesemann E. (2018). Global economic burden of diabetes in adults: projections from 2015 to 2030. *Diabetes Care*.

[2] Australian Institute of Health and Welfare (2020). https://www.aihw.gov.au/reports/diabetes/diabetes/contents/how-many-australians-have-diabetes.

[3] Saeedi P., Petersohn I., Salpea P. (2019). Global and regional diabetes prevalence estimates for 2019 and projections for 2030 and 2045: Results from the International Diabetes Federation Diabetes Atlas. *Diabetes Research and Clinical Practice*.

[4] Dall T. M., Yang W. Y., Gillespie K. (2019). The economic burden of elevated blood glucose levels in 2017: diagnosed and undiagnosed diabetes, gestational diabetes mellitus, and prediabetes. *Diabetes Care*.

[5] Selvin E., Wang D., Lee A. K., Bergenstal R. M., Coresh J. (2017). Identifying trends in undiagnosed diabetes in US adults by using a confirmatory definition a cross-sectional study. *Annals of Internal Medicine*.

[6] Gregg E. W., Cadwell B. L., Cheng Y. J. (2004). Trends in the prevalence and ratio of diagnosed to undiagnosed diabetes according to obesity levels in the U.S. *Diabetes Care*.

[7] Lailler G., Piffaretti C., Fuentes S. (2020). Prevalence of prediabetes and undiagnosed type 2 diabetes in France: results from the national survey ESTEBAN, 2014-2016. *Diabetes Research and Clinical Practice*.

[8] Tabák A. G., Herder C., Rathmann W., Brunner E. J., Kivimäki M. (2012). Prediabetes: a state for diabetes development. *The Lancet*.

[9] Bansal N. (2015). Prediabetes diagnosis and treatment: a review. *World Journal of Diabetes*.

[10] Australian Bureau of Statistics (2013). Australian health survey: biomedical results for chronic diseases. Australian Bureau of Statistics. https://www.abs.gov.au/statistics/health/health-conditions-and-risks/australian-health-survey-biomedical-results-chronic-diseases/latest-release#data-download.

[11] American Diabetes Association (2021). Standards of medical care in diabetes. *Diabetes Care*.

[12] Cowie C. C. (2019). Diabetes diagnosis and control: missed opportunities to improve health: the 2018 Kelly West Award Lecture. *Diabetes Care*.

[13] Shimodaira M., Okaniwa S., Hanyu N., Nakayama T. (2017). Optimal hemoglobin A1c levels for screening of diabetes and prediabetes in the Japanese population. *Journal Diabetes Research*.

[14] The Royal Australian College of General Practitioners (2016). *Guidelines for Preventive Activities in General Practice*.

[15] Sainsbury E., Shi Y., Flack J., Colagiuri S. (2018). *Burden of Diabetes in Australia Its Time for More Action Report*.

[16] Bell K., Shaw J. E., Maple-Brown L. (2020). A position statement on screening and management of prediabetes in adults in primary care in Australia. *Diabetes research and clinical practice*.

[17] The Royal Australian College of General Practitioners (2016). *General practice management of type 2 diabetes 2016-18*.

[18] Dhippayom T., Chaiyakunapruk N., Krass I. (2014). How diabetes risk assessment tools are implemented in practice: a systematic review. *Diabetes research and clinical practice*.

[19] Peer N., Balakrishna Y., Durao S. (2020). Screening for type 2 diabetes mellitus. *Cochrane Database of Systematic Reviews*.

[20] Chen L., Magliano D. J., Balkau B. (2010). AUSDRISK: an Australian Type 2 Diabetes Risk Assessment Tool based on demographic, lifestyle and simple anthropometric measures. *Medical Journal of Australia*.

[21] Australian Bureau of Statistics (2020). *Patient experiences in Australia: summary of findings*.

[22] Longato E., Di Camillo B., Sparacino G., Saccavini C., Avogaro A., Fadini G. P. (2020). Diabetes diagnosis from administrative claims and estimation of the true prevalence of diabetes among 4.2 million individuals of the Veneto region (North East Italy). *Nutrition, Metabolism, and Cardiovascular Diseases*.

[23] Tu K., Manuel D., Lam K., Kavanagh D., Mitiku T. F., Guo H. (2011). Diabetics can be identified in an electronic medical record using laboratory tests and prescriptions. *Journal of Clinical Epidemiology*.

[24] Imai C., Hardie R. A., Franco G. S. (2020). Harnessing the potential of electronic general practice pathology data in Australia: an examination of the quality use of pathology for type 2 diabetes patients. *International Journal of Medical Informatics*.

[25] Havard A., Manski-Nankervis J. A., Thistlethwaite J. (2021). Validity of algorithms for identifying five chronic conditions in MedicineInsight, an Australian national general practice database. *BMC Health Services Research*.

[26] Manski-Nankervis J. A., Thuraisingam S., Sluggett J. K. (2019). Prescribing of diabetes medications to people with type 2 diabetes and chronic kidney disease: a national cross-sectional study. *BMC Family Practice*.

[27] Manski-Nankervis J. E., Thuraisingam S., Lau P. (2018). Screening and diagnosis of chronic kidney disease in people with type 2 diabetes attending Australian general practice. *Australian Journal of Primary Health*.

[28] Bayram C., Britt H., Miller G., Valenti L. (2009). Evidence-Practice Gap in GP Pathology Test Ordering: A Comparison of BEACH Pathology Data and Recommended Testing. *Bettering the Evaluation And Care of Health*.

[29] Chiang J. I., Furler J., Mair F. (2020). Associations between multimorbidity and glycaemia (HbA1c) in people with type 2 diabetes: cross-sectional study in Australian general practice. *BMJ Open*.

[30] Roseleur J., Gonzalez-Chica D. A., Bernardo C. O., Geisler B. P., Karnon J., Stocks N. P. (2021). Blood pressure control in Australian general practice. *Journal of Hypertension.*.

[31] Harrison C., Henderson J., Miller G., Britt H. (2017). The prevalence of diagnosed chronic conditions and multimorbidity in Australia: a method for estimating population prevalence from general practice patient encounter data. *PLoS One*.

[32] NPS Medicine Wise (2020). *General practice insights report July 2018-June 2019*.

[33] Busingye D., Gianacas C., Pollack A. (2019). Data resource profile: MedicineInsight, an Australian national primary health care database. *International journal of epidemiology*.

[34] Horsfall L., Walters K., Petersen I. (2013). Identifying periods of acceptable computer usage in primary care research databases. *Pharmacoepidemiology and Drug Safety*.

[35] Benchimol E. I., Smeeth L., Guttmann A. (2015). The REporting of studies Conducted using Observational Routinely-collected health Data (RECORD) statement. *PLoS Medicine*.

[36] The Royal Australian College of General Practitioners (2015). *The RACGP Standards for general practices*.

[37] Australian Bureau of Statistics (2018). *Census of Population and Housing: Socio-Economic Indexes for Areas (SEIFA), Australia. Cat. No. 2033.0.55.001*.

[38] Australian Bureau of Statistics *National Health Survey: first results. Presents key findings for health statistics including long-term health conditions; mental wellbeing; and health risk factors. Canberra 2018*.

[39] Kiefer M. M., Silverman J. B., Young B. A., Nelson K. M. (2015). National patterns in diabetes screening: data from the National Health and Nutrition Examination Survey (NHANES) 2005-2012. *Journal of General Internal Medicine*.

[40] Gonzalez-Chica D. A., Bowden J., Miller C. (2019). Patient-reported GP health assessments rather than individual cardiovascular risk burden are associated with the engagement in lifestyle changes: population-based survey in South Australia. *BMC Family Practice*.

[41] Li Y. R., Zhao L. Y., Yu D. M., Ding G. (2018). The prevalence and risk factors of dyslipidemia in different diabetic progression stages among middle-aged and elderly populations in China. *PLoS One*.

[42] Zhang Y. Y., Liu Y. Y., Su Y. Y. (2017). The metabolic side effects of 12 antipsychotic drugs used for the treatment of schizophrenia on glucose: a network meta-analysis. *BMC Psychiatry*.

[43] Mangurian C., Newcomer J. W., Vittinghoff E. (2015). Diabetes screening among underserved adults with severe mental illness who take antipsychotic medications. *JAMA Internal Medicine*.

[44] Greiver M., Aliarzadeh B., Moineddin R., Meaney C., Ivers N. (2011). Diabetes screening with hemoglobin A1c prior to a change in guideline recommendations: prevalence and patient characteristics. *BMC Family Practice*.

[45] Britt H., Miller G. C., Henderson J. (2016). *General Practice Activity in Australia 2015-16*.

[46] Yousaf O., Grunfeld E. A., Hunter M. S. (2015). A systematic review of the factors associated with delays in medical and psychological help-seeking among men. *Health Psychology Review*.

[47] Si S., Moss J., Karnon J., Stocks N. (2018). Cost-effectiveness evaluation of the 45-49 year old health check versus usual care in Australian general practice: a modelling study. *PLoS One*.

