# Supplementary material for: Diabetes Mellitus Diagnosis and Screening in Australian General Practice: A National Study
Source: J Diabetes Res. 2022 Mar 23;2022:1566408. doi: 10.1155/2022/1566408 (PMC8968388; doi:10.1155/2022/1566408)
Supplement: Supplementary 1 — Supplementary Table 1: definitions of recorded diabetes, recorded prediabetes, and unrecorded diabetes/prediabetes. [file 1566408.f1.docx]

Supplementary Table 1. Definitions of recorded diabetes, recorded prediabetes, and unrecorded diabetes/prediabetes

| Outcomes | Definitions |
| --- | --- |
| A) Recorded diabetes | (1) Have a diagnosis of ‘diabetes mellitus’ in two fields (either in the diagnosis, reason for encounter, or reason for prescription fields) or in two different occasions in the same field, OR;  (2) They were prescribed insulin (ATC code A10A) AND/OR an oral antidiabetic medication (ATC code A10B, excepted metformin): glibenclamide, gliclazide, glimepiride, glipizide, acarbose, pioglitazone, alogliptin, linagliptin, saxagliptin, sitagliptin, vildagliptin, dulaglutide, exenatide, dapagliflozin, empagliflozin, ertugliflozin, OR;  (3a) Have a diagnosis of ‘diabetes mellitus’ in one field (either in the diagnosis, reason for encounter, or reason for prescription fields), AND were prescribed metformin (in absence of PCOS diagnosis);  (3b) Have a diagnosis of ‘diabetes mellitus’ in one field only (either in the diagnosis, reason for encounter, or reason for prescription fields), AND have one recorded laboratory test with raised glucose levels within the last 24 months [i.e. fasting plasma glucose ≥7.0 mmol/L; random plasma glucose ≥11.1 mmol/L; HbA1c ≥48 mmol/mol (≥6.5%)] or a OGTT ≥11.1 mmol/L. |
| B) Unrecorded diabetes | (1) They do not satisfy the criteria presented in “A” for diagnosed diabetes, AND;  (2) Have at least two recorded laboratory tests with raised glucose levels within 24 months [i.e. fasting plasma glucose ≥ 7.0 mmol/L; random plasma glucose ≥11.1 mmol/L; HbA1c ≥48 mmol/mol (≥6.5%)] or a OGTT ≥11.1 mmol/L.  Note: If the patient had only one altered laboratory test compatible with diabetes, but no ‘diabetes diagnosis’ or a prescription for diabetes, they will be classified and reported as an ‘incomplete diagnosis (insufficient data)’. |
| C) Recorded prediabetes | (1) They do not satisfy the criteria presented in “A” or “B” for diabetes, AND;  (2) Have a diagnosis of ‘prediabetes’ in two fields (either in the diagnosis, reason for encounter, or reason for prescription fields) or in two different occasions in the same field, OR;  (3a) Have a diagnosis of ‘prediabetes’ in one field (either in the diagnosis, reason for encounter, or reason for prescription fields), AND were prescribed metformin (in absence of PCOS diagnosis);  (3b) Have a diagnosis of ‘prediabetes’ in one field only (either in the diagnosis, reason for encounter, or reason for prescription fields), AND have one recorded laboratory test indicating prediabetes within the last 24 months [i.e. fasting blood glucose 6.1-6.9 mmol/L and/or a OGTT is between 7.8-11.1 mmol/L OR HbA1c between 42 and 47 mmol/mol (6.0%-6.4%)] |
| D) Unrecorded prediabetes | (1) They do not satisfy the criteria presented in “C” for diagnosed prediabetes, AND;  (2) Have at least two laboratory results recorded within the last 24 months indicating prediabetes (i.e. fasting blood glucose 6.1-6.9 mmol/L and/or OGTT is between 7.8-11.1 mmol/L OR HbA1c between 42 and 47 mmol/mol (6.0%-6.4%)]  Note: If the patient had only one altered laboratory test compatible with prediabetes and no prediabetes diagnosis, they will be classified and reported as ‘incomplete diagnosis (insufficient data)’. |

ATC: Anatomical Therapeutic Chemical Classification; PCOS: Polycystic ovary syndrome: HbA1c: Hemoglobin A1C; OGTT: 2-hour oral glucose tolerance; Study period is from 2016 to 2018.
