# Supplementary material for: Diabetes Mellitus Diagnosis and Screening in Australian General Practice: A National Study
Source: J Diabetes Res. 2022 Mar 23;2022:1566408. doi: 10.1155/2022/1566408 (PMC8968388; doi:10.1155/2022/1566408)
Supplement: Supplementary 2 — Supplementary Table 2: calculation of the Australian Type 2 Diabetes Risk (AUSDRISK) Assessment Tool score using variables available in the MedicineInsight database. [file 1566408.f2.docx]

Supplementary Table 2. Calculation of The Australian Type 2 Diabetes Risk Assessment Tool (AUSDRISK) score using variables available in the MedicineInsight database

| Variables in MedicineInsight | Categories and points |
| --- | --- |
| Age | - Under 35 years [0 points] - 35 - 44 years [2 points] - 45 - 54 years [4 points] - 55 - 64 years [6 points] - 65 - 109 years [8 points] |
| Gender | - Female [0 points] - Male [3 points] - Not recorded [0 points] |
| Aboriginal/Torres Strait Islander | - No [0 points] - Yes [2 points] - Not recorded [0 points] |
| High blood pressure | - No record [0 points] - Yes (at least one record) [6 points] |
| Antidiabetic medication | - No record [0 points] - Yes (at least one record) [2 points] |
| Smoking status | - Non-smoker [0 points] - Ex-smoker [0 points] - Smoker [2 points] - Not recorded [0 points] |
| Total points | **The sum of each variable, totalling 0 to 23** |
