# Supplementary material for: Diabetes Mellitus Diagnosis and Screening in Australian General Practice: A National Study
Source: J Diabetes Res. 2022 Mar 23;2022:1566408. doi: 10.1155/2022/1566408 (PMC8968388; doi:10.1155/2022/1566408)
Supplement: Supplementary 3 — Supplementary Table 3: sociodemographic profile of the study population (regular patients aged 18+ years) with 95% CI according to diabetes diagnosis status (2016-2018). [file 1566408.f3.docx]

Supplementary Table 3. Sociodemographic profile of the study population (regular patients aged 18+ years) with 95%CI according to diabetes diagnosis status (2016-2018)

| Characteristics | All regular patients, aged 18+ years | | Recorded diabetes | | Recorded prediabetes | | Unrecorded diabetes/prediabetes | |
| --- | --- | --- | --- | --- | --- | --- | --- | --- |
|  | n | %(95%CI) | n | %(95%CI) | n | %(95%CI) | n | %(95%CI) |
| Gender |  |  |  |  |  |  |  |  |
| Male | 628,040 | 41.9(41.4-42.3) | 75,976 | 52.2 (51.6-52.9) | 6,485 | 54.8(53.1-56.5) | 2,713 | 53.7(52.1-55.2) |
| Female | 893,541 | 58.1(57.7-58.6) | 69,762 | 47.8(47.1-48.4) | 5,566 | 45.2(43.5-46.9) | 2,398 | 46.3(44.8-47.9) |
| Age group |  |  |  |  |  |  |  |  |
| 18-29 | 220,747 | 17.9(17.2-18.7) | 3,689 | 3.1(2.9-3.4) ^a**^ | 157 | 1.5(1.2-1.9) ^b**^ | 20 | 0.5(0.3-0.8) |
| 30-39 | 224,571 | 17.1(16.4-17.8) | 6,863 | 5.6(5.2-6.0) ^a**^ | 633 | 6.2(5.3-7.2) ^b**^ | 116 | 2.8(2.2-3.6) |
| 40-49 | 236,642 | 16.1(15.7-16.4) | 13,036 | 9.7(9.2-10.1) ^a**^ | 1,511 | 13.6(12.4-14.7) ^b**^ | 240 | 5.4(4.7-6.3) |
| 50-59 | 253,499 | 16.0(15.8-16.3) | 24,218 | 17.1(16.7-17.6) ^a**^ | 2,749 | 23.8(22.8-24.9) ^b**^ | 673 | 14.0(13.0-15.1) |
| 60-69 | 255,565 | 15.1(14.6-15.5) | 37,558 | 25.6(25.2-26.0) ^a*^ | 3,596 | 29.4(28.3-30.6) | 1,378 | 27.5(26.2-28.9) |
| 70-79 | 204,651 | 11.2(10.6-11.7) | 38,140 | 24.8(24.1-25.4) ^a**^ | 2,563 | 19.5(18.1-20.9) ^b**^ | 1,572 | 29.6(28.3-31.0) |
| 80-89 | 100,831 | 5.5(5.2-5.8) | 18,938 | 12.4(11.9-12.9) ^a**^ | 753 | 5.6(5.0-6.2) ^b**^ | 921 | 17.1(15.9-18.4) |
| 90+ | 20,461 | 1.1(1.0-1.2) | 2,476 | 1.7(1.6-1.8) ^a**^ | 58 | 0.4(0.3-0.6) ^b**^ | 144 | 3.0(2.4-3.7) |
| Practice remoteness |  |  |  |  |  |  |  |  |
| Major Cities | 949,538 | 64.5(59.8-68.8) | 85,695 | 60.3(55.3-65.0) | 7,611 | 64.5(58.4-70.1) | 2,889 | 57.9(51.9-63.6) |
| Inner regional | 377,329 | 23.5(19.9-27.5) | 39,506 | 26.2(22.2-30.7) | 2,949 | 23.7(19.1-29.1) | 1,437 | 27.2(22.2-32.9) |
| Outer regional/Remote | 188,482 | 12.0(9.6-15.1) | 19,877 | 13.5(10.7-16.8) | 1,439 | 11.8(8.8-15.7) | 764 | 14.9(11.6-19.0) |
| Practice IRSAD quintile |  |  |  |  |  |  |  |  |
| Very high | 380,447 | 25.3(22.1-28.8) | 27,275 | 19.1(16.4-22.0) ^a**^ | 2,757 | 23.0(19.2-27.3) | 1,170 | 23.1(19.3-27.4) |
| High | 283,925 | 19.4(17.2-21.8) | 24,121 | 17.0(14.9-19.3) | 2,282 | 19.3(16.3-22.7) | 859 | 17.3(14.9-20.0) |
| Middle | 351,266 | 22.8(20.1-25.8) | 36,259 | 24.6(21.5-27.9) | 2,822 | 23.2(19.5-27.5) | 1,195 | 23.1(19.4-27.2) |
| Low | 249,024 | 16.3(14.0-18.8) | 26,979 | 18.3(15.8-21.1) | 1,982 | 16.2(13.1-20.0) | 798 | 15.9(13.1-19.1) |
| Very low | 246,478 | 15.5(13.0-18.3) | 30,098 | 20.3(17.2-23.8) | 2,130 | 17.6(14.1-21.8) | 1,062 | 20.1(16.3-24.5) |

IRSAD: Index of Relative Socioeconomic Advantage and Disadvantage. A higher score indicating the practice is located in a more advantaged area. (a) P-value for the difference between people with recorded diabetes and unrecorded diabetes/prediabetes; (b) P-value for the difference between people with recorded prediabetes and unreported diabetes/prediabetes; P-value *<0.01, **<0.001; (c) Practice IRSAD quintile includes 0.8% of missing data.
