# Supplementary material for: Diabetes Mellitus Diagnosis and Screening in Australian General Practice: A National Study
Source: J Diabetes Res. 2022 Mar 23;2022:1566408. doi: 10.1155/2022/1566408 (PMC8968388; doi:10.1155/2022/1566408)
Supplement: Supplementary 4 — Supplementary Figure 1: prevalence of recorded diabetes (A), recorded prediabetes (B), and unrecorded diabetes/prediabetes (C) among all adults aged 18+ years, by age group and gender, Australia, 2016-2018. [file 1566408.f4.docx]

Supplementary Figure 1. Prevalence of recorded diabetes (A), recorded prediabetes (B), and unrecorded diabetes/prediabetes (C) among all adults aged 18+ years, by age group and gender, Australia, 2016-2018

(A)

（B）

(C)
