# Supplementary material for: Diabetes Mellitus Diagnosis and Screening in Australian General Practice: A National Study
Source: J Diabetes Res. 2022 Mar 23;2022:1566408. doi: 10.1155/2022/1566408 (PMC8968388; doi:10.1155/2022/1566408)
Supplement: Supplementary 5 — Supplementary Figure 2: proportion of recorded diabetes, recorded prediabetes, and unrecorded diabetes/prediabetes among regular patients aged 18+ years, by gender (A), age (B), practice remoteness (C), and practice IRSAD (D), Australia, 2016-2018. [file 1566408.f5.docx]

Supplementary Figure 2. Proportion of recorded diabetes, recorded prediabetes, and unrecorded diabetes/prediabetes among regular patients aged 18+ years, by gender (A), age (B), practice remoteness (C), and practice IRSAD (D). Australia, 2016-2018

（A） (B)

（C） (D)
